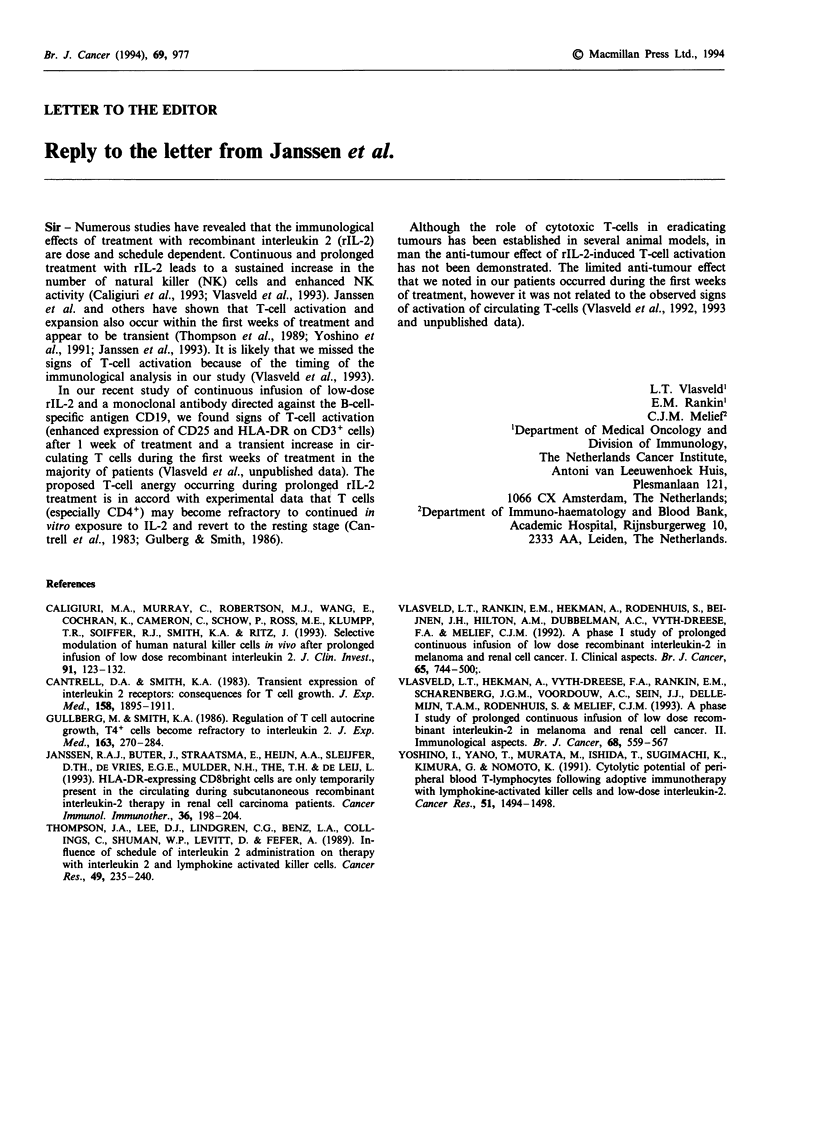# Reply to the letter from Janssen et al

**Published:** 1994-05

**Authors:** L.T. Vlasveld, E.M. Rankin, C.J.M. Melief


Br. J. Cancer (1994), 69, 977 © Macmillan Press Ltd., 1994

LETTER TO THE EDITOR

### Reply to the letter from Janssen *et al.*

**Sir** – Numerous studies have revealed that the immunological effects of treatment with recombinant interleukin 2 (rIL-2) are dose and schedule dependent. Continuous and prolonged treatment with rIL-2 leads to a sustained increase in the number of natural killer (NK) cells and enhanced NK activity (Caligiuri *et al.*, 1993; Vlasveld *et al.*, 1993). Janssen *et al.* and others have shown that T-cell activation and expansion also occur within the first weeks of treatment and appear to be transient (Thompson *et al.*, 1989; Yoshino *et al.*, 1991; Janssen *et al.*, 1993). It is likely that we missed the signs of T-cell activation because of the timing of the immunological analysis in our study (Vlasveld *et al.*, 1993).

In our recent study of continuous infusion of low-dose rIL-2 and a monoclonal antibody directed against the B-cell-specific antigen CD19, we found signs of T-cell activation (enhanced expression of CD25 and HLA-DR on $CD3^+$ cells) after 1 week of treatment and a transient increase in circulating T cells during the first weeks of treatment in the majority of patients (Vlasveld *et al.*, unpublished data). The proposed T-cell anergy occurring during prolonged rIL-2 treatment is in accord with experimental data that T cells (especially $CD4^+$) may become refractory to continued *in vitro* exposure to IL-2 and revert to the resting stage (Cantrell *et al.*, 1983; Gulberg & Smith, 1986).

Although the role of cytotoxic T-cells in eradicating tumours has been established in several animal models, in man the anti-tumour effect of rIL-2-induced T-cell activation has not been demonstrated. The limited anti-tumour effect that we noted in our patients occurred during the first weeks of treatment, however it was not related to the observed signs of activation of circulating T-cells (Vlasveld *et al.*, 1992, 1993 and unpublished data).

L.T. Vlasveld[1]
E.M. Rankin[1]
C.J.M. Melief[2]
[1]Department of Medical Oncology and
Division of Immunology,
The Netherlands Cancer Institute,
Antoni van Leeuwenhoek Huis,
Plesmanlaan 121,
1066 CX Amsterdam, The Netherlands;
[2]Department of Immuno-haematology and Blood Bank,
Academic Hospital, Rijnsburgerweg 10,
2333 AA, Leiden, The Netherlands.

### References

CALIGIURI, M.A., MURRAY, C., ROBERTSON, M.J., WANG, E., COCHRAN, K., CAMERON, C., SCHOW, P., ROSS, M.E., KLUMPP, T.R., SOIFFER, R.J., SMITH, K.A. & RITZ, J. (1993). Selective modulation of human natural killer cells *in vivo* after prolonged infusion of low dose recombinant interleukin 2. *J. Clin. Invest.*, **91**, 123–132.

CANTRELL, D.A. & SMITH, K.A. (1983). Transient expression of interleukin 2 receptors: consequences for T cell growth. *J. Exp. Med.*, **158**, 1895–1911.

GULLBERG, M. & SMITH, K.A. (1986). Regulation of T cell autocrine growth, $T4^+$ cells become refractory to interleukin 2. *J. Exp. Med.*, **163**, 270–284.

JANSSEN, R.A.J., BUTER, J., STRAATSMA, E., HEIJN, A.A., SLEIJFER, D.TH., DE VRIES, E.G.E., MULDER, N.H., THE, T.H. & DE LEIJ, L. (1993). HLA-DR-expressing CD8bright cells are only temporarily present in the circulating during subcutanoneous recombinant interleukin-2 therapy in renal cell carcinoma patients. *Cancer Immunol. Immunother.*, **36**, 198–204.

THOMPSON, J.A., LEE, D.J., LINDGREN, C.G., BENZ, L.A., COLLINGS, C., SHUMAN, W.P., LEVITT, D. & FEFER, A. (1989). Influence of schedule of interleukin 2 administration on therapy with interleukin 2 and lymphokine activated killer cells. *Cancer Res.*, **49**, 235–240.

VLASVELD, L.T., RANKIN, E.M., HEKMAN, A., RODENHUIS, S., BEIJNEN, J.H., HILTON, A.M., DUBBELMAN, A.C., VYTH-DREESE, F.A. & MELIEF, C.J.M. (1992). A phase I study of prolonged continuous infusion of low dose recombinant interleukin-2 in melanoma and renal cell cancer. I. Clinical aspects. *Br. J. Cancer*, **65**, 744–500;.

VLASVELD, L.T., HEKMAN, A., VYTH-DREESE, F.A., RANKIN, E.M., SCHARENBERG, J.G.M., VOORDOUW, A.C., SEIN, J.J., DELLEMIJN, T.A.M., RODENHUIS, S. & MELIEF, C.J.M. (1993). A phase I study of prolonged continuous infusion of low dose recombinant interleukin-2 in melanoma and renal cell cancer. II. Immunological aspects. *Br. J. Cancer*, **68**, 559–567

YOSHINO, I., YANO, T., MURATA, M., ISHIDA, T., SUGIMACHI, K., KIMURA, G. & NOMOTO, K. (1991). Cytolytic potential of peripheral blood T-lymphocytes following adoptive immunotherapy with lymphokine-activated killer cells and low-dose interleukin-2. *Cancer Res.*, **51**, 1494–1498.